# P05.33. Becoming aware of your body: a qualitative study on yoga for chronic neck pain patients

**DOI:** 10.1186/1472-6882-12-S1-P393

**Published:** 2012-06-12

**Authors:** H Cramer, R Lauche, H Haller, J Langhorst, G Dobos, B Berger

**Affiliations:** 1 University of Duisburg, Complementary and Integrative Medicine, Essen, Germany; 2 University of Witten-Herdecke, Gerhard Kienle Chair, Herdecke, Germany

## Purpose

To investigate perceived changes in body perception and psychosocial aspects in chronic neck pain patients after participating in a yoga program.

## Methods

Eighteen patients with chronic non-specific neck pain participated in a 9-week Iyengar yoga-program. Before and after the program, patients were asked to complete a drawing of their neck and shoulder region in a way that reflects their subjective body perception. Semi-standardized interviews were used to retrieve more information on body perception, emotional status, everyday life and coping, and changes in these dimensions after attendance in the program. An interdisciplinary interpretation group analyzed the interviews using the content analysis approach according to Mayring.

## Results

Patients reported changes on 5 fundamental dimensions of human experience: the physical, cognitive, emotional, behavioral and social dimensions. On the physical dimension patients mainly reported a renewed body awareness and body mindfulness. This was also obvious in the body drawings that were distorted and incomplete before the yoga program and normalized after attendance of the program. Patients further described changes on the cognitive dimension, mainly increased perceived control over their health and cognitive reappraisal of physical activity, and on the emotional dimension, particularly acceptance of their pain and life’s burden. On the behavioral dimension patients reported the enhanced use of active coping strategies, and on the social dimension patients particularly described a renewed participation in active life.

## Conclusion

Yoga induced changes on a wide range of experiential dimensions. Patients perceived yoga as helpful in coping with their pain, gaining more control over their health and well-being and increasing pain acceptance. Body awareness seems to be a key mechanism of these changes.

